# Managing and Retrieving Bilingual Documents Using Artificial Intelligence-Based Ontological Framework

**DOI:** 10.1155/2022/4636931

**Published:** 2022-08-25

**Authors:** Abdulaziz Fahad Alothman, Abdul Rahaman Wahab Sait

**Affiliations:** Department of Documents and Archive, Center of Documents and Administrative Communication, King Faisal University, P.O. Box 400, Al Hofuf 31982, Al-Ahsa, Saudi Arabia

## Abstract

In recent times, artificial intelligence (AI) methods have been applied in document and content management to make decisions and improve the organization's functionalities. However, the lack of semantics and restricted metadata hinders the current document management technique from achieving a better outcome. E-Government activities demand a sophisticated approach to handle a large corpus of data and produce valuable insights. There is a lack of methods to manage and retrieve bilingual (Arabic and English) documents. Therefore, the study aims to develop an ontology-based AI framework for managing documents. A testbed is employed to simulate the existing and proposed framework for the performance evaluation. Initially, a data extraction methodology is utilized to extract Arabic and English content from 77 documents. Researchers developed a bilingual dictionary to teach the proposed information retrieval technique. A classifier based on the Naïve Bayes approach is designed to identify the documents' relations. Finally, a ranking approach based on link analysis is used for ranking the documents according to the users' queries. The benchmark evaluation metrics are applied to measure the performance of the proposed ontological framework. The findings suggest that the proposed framework offers supreme results and outperforms the existing framework.

## 1. Introduction

The recent development in the information retrieval (IR) techniques facilitates effective document management (DM) functionalities in organizations. The process of retrieving relevant information by passing a query in a search engine is called IR [1–5]. A query is a text in a natural language to extract a relevant document. For instance, a search engine can fetch approximately one million webpages for a user query. Organizations apply business intelligence (BI) tools to process a large amount of data and retrieve valuable information [6–11]. To compete effectively, organizations should analyze and leverage a wide range of data, information, and expertise in order to make effective decisions. Decision Support Systems (DSS) are interactive computer-based systems designed to assist decision-makers in identifying and solving problems, completing decision process tasks, and making decisions [12–19]. These systems are becoming increasingly popular among managers due to this trend. However, the shortcomings include unstructured data and complex queries reducing IR technologies' performance. In other words, users failed to retrieve relevant documents for their queries [20–25]. Moreover, the absence of bilingual (English and Arabic) IR systems causes difficulties for organizations in the Middle East countries.

On the one hand, there is an availability of a wide range of IR systems. On the other hand, there is a lack of domain-specific ontologies or IR systems to serve an organization [26–30]. In the Kingdom of Saudi Arabia (KSA), most organizations offer a sophisticated application for employees and stakeholders to share the information and valuable documents. The internal communication between employees generates a larger amount of documents [31–35]. Organizations demand an artificial intelligence (AI) based system to generate knowledge from the documents [36–41].

In the current environment, organizations store documents in Portable Document Format (PDF) form and their relevant metadata in a different storage location. The AI tools widely use the metadata for making decisions [42–47]. There are many techniques for retrieving a document using a query. Thus, organizations cannot access the document's content without its metadata [48–51]. The KSA's Vision 2030 motivates researchers to apply innovative techniques to the current functionalities of the organization. Therefore, developing an ontological framework for document management can support organizations in satisfying their stakeholders. In addition, the role of natural language processing (NLP) in the ontological framework enables individuals to interact with the system in their natural language [52–54].

The objectives of the study are:

1. Build a data extraction model for extracting text from Arabic and English PDF documents.
2. Construct a name entity-relationship (NER) classifier for classifying the documents.
3. Implement a ranking approach to retrieve relevant documents for a user query.

The remaining part of the study is organized as follows: Section 2 reports the features of existing literature and research gaps.  Section 3 outlines the research methodology and Section 4 discusses the study's findings. Finally, Section 5 concludes the study with its future direction.

## 2. Literature Review

DM is one of the critical processes in an organization. The communication between the users of the internal and the external units of an organization may generate a document [1–5]. Organizations follow the government and the international archival policies to store and manage their documents [6–9]. The existing studies show many techniques and frameworks for managing documents and IR [9–15].

Zaman et al. proposed an ontological framework for retrieving scientific sources [1]. They employed fuzzy rule base and word sense disambiguation for extracting information from multiple scientific documents. The experimental outcome suggests that the framework was less sensitive to the document file format modifications. However, there is limited information on the performance of the framework.

Yao et al. developed an AI-based ontological model for predicting the side effects of medicines [2]. The model had certain entities such as value and relationships. The value and relationship are used to indicate the drug and its side effects. The AI model's fuzzy and dynamically defined latent attributions can redefine vital records. The performance of the IR model is affected by the limitations, including the lack of negative data and the smaller dataset.

Crimp and Trotman proposed a linguistic model using Roget's and WordNet [3]. They employed an Attre search engine and evaluated the model using the mean average precision (MAP) metric. The outcome highlights the better performance of the linguistic model. However, the authors utilized a limited set of features from Roget's and WordNet.

Vocabulary mismatch is one of the limitations of the IR system. To overcome this limitation, query expansion (QE) techniques are developed. However, QE techniques are based on specialization and context relationships [4]. Raza et al. discussed that domain-specific ontologies are widely used in medicine, agriculture, and other scientific fields [4]. Multiple automated QE systems are proposed in IR [5]. Yunzhi et al. constructed an Arabic ontology based on the Protégé and SPARQL language to extract candidate expansion terms [6].

Domain-independent ontologies serve as a valuable resource for multiple domains. Aggarwal and Paul extracted expansion concepts from DBPedia and Wikipedia ontologies using semantic analysis [7]. However, the shortcomings include ambiguous terms and a lack of unique ontological properties causes more complexities. Zingla et al. and Omar et al. proposed hybrid models for extracting expansion concepts from DBpedia and Wikipedia [8, 9]. They employed Microblog and TREC 2011 datasets for evaluating their ontological performance.

The existing studies focus on the specific domains, and there are no studies on the DM and IR [10–15]. There is a lack of bilingual ontological framework for the organizations in the KSA. Most studies considered the NER classification of webpages as a primary objective rather than the ranking approach [16–21]. Particle Swarm Optimization (PSO) is used to enhance and train Hidden Markov Model (HMM) estimate approaches (PSO). PSO identifies the optimal response for a user query. For instance, the metadata of a document can be extracted using this approach [22–27]. A text extractor can be built using the AI technique for the automated extraction of key terms from a document [28–34]. An ontology-based dynamic information extraction framework identifies a wide range of document resources published in the scientific community and extracts the whole structural information [35–41]. The accuracy and scope of information extraction can be improved using an entity-relationship-based framework [42–47]. Few research works employed the term—frequency methodology for ranking the webpages [48–54]. Thus, there is a demand for a practical ontological framework for managing documents and retrieving information based on the user query. Furthermore, the recent ontological frameworks, including Gohar Zaman et al. (GOF) and Yuazhe Yao et al. (YOF), are employed to compare the performance of the proposed ontological framework (POF).

## 3. Research Methodology

**Figure 1:**
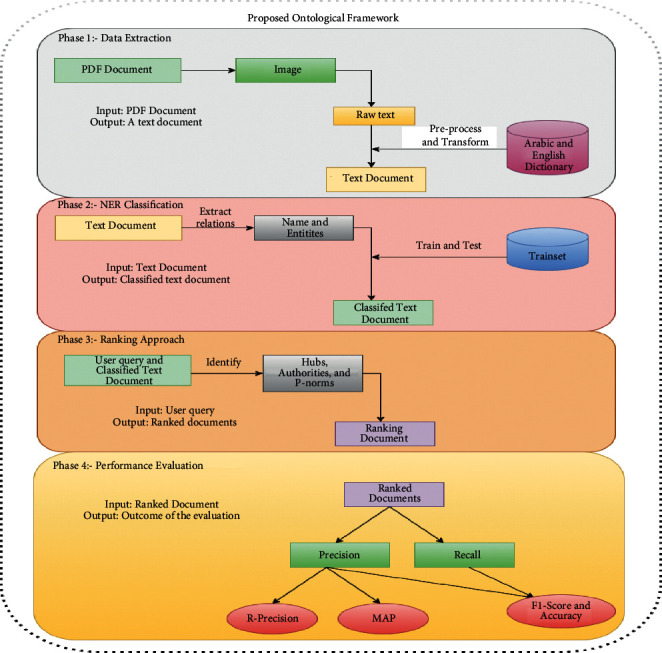
Proposed research framework.

In order to achieve the objective of the study, researchers construct a bilingual (Arabic and English) ontological framework for retrieving documents.  Figure 1 presents the proposed research framework of the proposed study. It covers four phases including data extraction, NER classification, ranking technique, and performance evaluation.

The first phase outlines the data extraction process for extracting text from PDF documents. The NER classification using MNB is described in the second phase. The third phase highlights the ranking techniques to retrieve relevant documents. Lastly, the fourth phase evaluates the performance of the proposed ontological framework (POF).

### 3.1. Phase 1: Data Extraction

**Figure 2:**
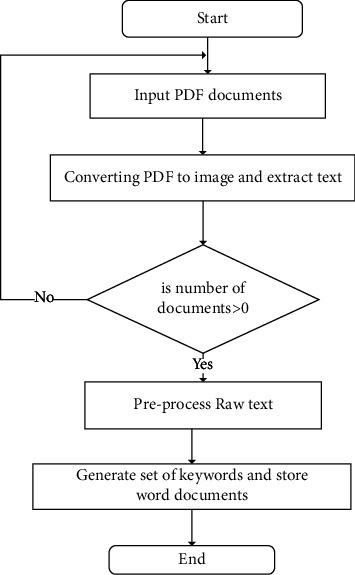
Text extraction process.

This phase transforms the PDF document into a text document. It supports the retrieval process to extract relevant documents. During communication, employees or stakeholders widely use PDF documents for sharing information. It is difficult to search a PDF document using a user query. Therefore, A PDFtoWord is developed in order to automate the process of converting a PDF document to a Word document. However, a PDF document may contain handwritten content which cannot be converted into a Word document. In other words, converting handwritten text into standard text is challenging.  Figure 2 shows the activities of phase 1. Initially, a document is converted to image format in order to extract the text. The extracted raw text is preprocessed and stored as a set of keywords and a word file. Phase 1 supports the proposed framework to search a document using a keyword. It overcomes the limitations of the searching document using metadata.

Thus, this study transforms the PDF document into an image, JPEG, or PNG format. The procedure of the data extraction process is as follows:

Step 1: Input a PDF document.
Step 2: Converting documents from a PDF form to JPEG or PNG format.
Let PD be the PDF document, ID be an image format of the PDF document. Doc_To_Img is a function for converting the documents from PDF to image structure and hres is the attribute to make the image with high resolution (1100 × 900 pixels at 600 pixels per inch). Equation (1) shows the expression of converting the PDF document into image format.
  (1) $$\begin{array}{c} \text{ID }=\text{Doc\_To\_Img}\left(\text{PD}\right)\cdot \text{hres}\left(1100\text{,}900\text{,}600\right). \end{array}$$
Step 3: Designing a text extractor.

A text extractor is designed using the AI-based Tessaract module that extracts the text from the image [55]. Nonetheless, the module is limited to the English Language. Thus, a dedicated Arabic dictionary is developed and integrated with the Tessaract module. Let Tessaract() be a function to extract text from an image, P_process be a preprocess function, RT is a raw text, and *d* be the document's content. Equations (2) and (3) outline the extraction and preprocessing of text.

(2) $$\begin{array}{c} \text{RT}=\text{Tessaract}\left(\text{ID}\right), \end{array}$$

(3) $$\begin{array}{c} D=P\text{\_process}\left(\text{RT}\right). \end{array}$$

The P_process function employs an Arabic and English dictionary to ensure the RT is correct. During the text extraction, the extracted text may contain some errors. For instance, “name” may be misspelt as “mame.” Thus, the dictionary corrects the erroneous content.

### 3.2. Phase 2: NER Classification

**Figure 3:**
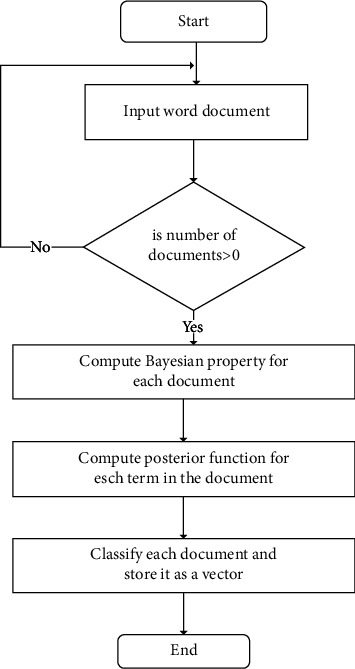
Entity–relationship classification.

In this proposed study, the researchers employed the Multinomial Naïve Bayes (MNB) for classifying the documents [56]. Each document is a collection of words. A class or label consists of homogeneous documents. MNB algorithm is widely used in NLP applications. It classifies documents based on the statistical outcome of the content.  Figure 3 outlines the processes of phase 2. The word document is processed using the Bayesian property. The posterior function is computed for each term in the document. Finally, each document is stored as a vector. The following section explains the computation of Bayesian property and posterior function in detail.

**Figure 4:**
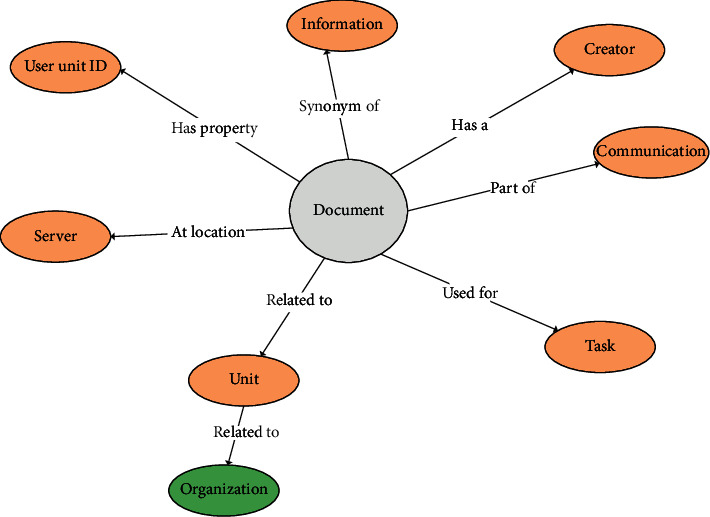
Document relationship.

**Figure 5:**
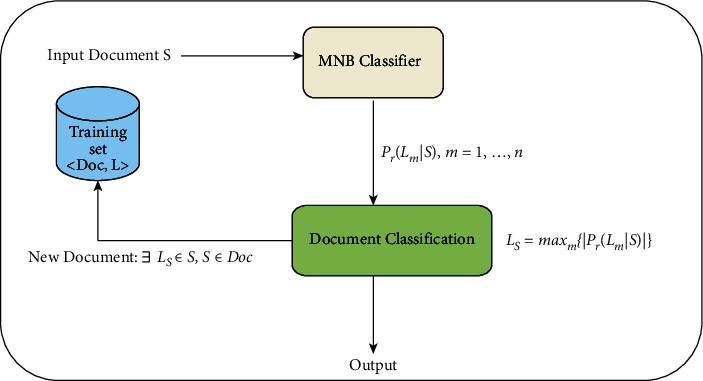
Document classification model [24].

The classification assigns a text segment to a class using the probability of documents in the class of other documents. The process of grouping similar documents under a specific class is called labeling. Let *S* be the document to be classified. Each document in *S* is treated as a string related to one or multiple documents based on a class *L*. The classification of documents is based on a train set that contains the classified documents according to the document relationship in Figure 4.  Figure 5 shows the classification of documents using the train set.

Let *f* be the vector in *S*, *f*_i_ be the feature in *f* representing the *i*^th^ term in *L*. The core of the MNB model is the evaluation of probability-based decision function. The Bayesian probability for the documents is expressed in equations (4) and (5). The likelihood of the *i*^th^ term *f*_*i*_ belonging to the class *L*_*m*_ is shown in equation (6). Equation (7) outlines the MNB in the log space. The evaluation log(*P*) is expressed in (8).

(4) $$\begin{array}{c} P\left(L_{m}|f\right)=\;\frac{P\left(L_{m}\right)XP\left(fL_{m}|\right)}{P\left(f\right)}, \end{array}$$

(5) $$\begin{array}{c} P_{r}\left(f|L_{m}\right)=\;\frac{\left(\sum_{i=1}^{n}f_{i}\right)!}{\left({\prod_{i=1}^{n}f}_{i}\right)!}X\prod_{i=1}^{n}p_{m_{i}}^{f_{i}}, \end{array}$$

(6) $$\begin{array}{c} P_{r}\left(f|L_{m}\right)=\prod_{i=1}^{n}P\left(f_{i}|L_{m}\right), \end{array}$$

(7) $$\begin{array}{c} \text{log}P_{r}\left(L_{m}|f\right)\propto \text{log}P\left(L_{m}\right)+\sum_{i=1}^{n}f_{i}X\text{log}P\left(f_{i}|L_{m}\right), \end{array}$$

(8) $$\begin{array}{c} \text{log }\left(P\right)=\left\{\begin{array}{cc} \text{ln}\left(P\right), & P<1,\\ 1.0, & P\leq 1. \end{array}\right. \end{array}$$

The following steps are followed for classifying the documents using the MNB classifier:

Step 1: Divide the documents (*S*) into a group of n-terms.
Step 2: Repeat the following process for each *i*^th^ term in *S*.
Step 2(a): Compute the Bayesian probability using equation (4).
Step 2(b): Evaluate the *P*(*Lm*) function for each document *i* in *L*.
Step 2(c): Compute the posterior function by integrating the prior function to the sum of each term using equation.
  (9) $$\begin{array}{c} P_{r}\left(L_{m}|f\right)=\text{log}P\left(L_{m}\right)+\sum_{i=1}^{n}\left[f_{i}*\text{log}P\left(f_{i}|L_{m}\right)\right]. \end{array}$$
Step 3: Compute *L*_*S*_ of *S* using Eqn.
  (10) $$\begin{array}{c} L_{S}=\;\begin{array}{c} \text{argMax}\\ m\in \;\left(1\ldots n\right) \end{array}\left|P_{r}\left(L_{m}|f\right)\right|. \end{array}$$
Step 4: Repeat Steps 1 to 3 with the train set.
Step 5: Classify the documents and store them as a vector.

### 3.3. Phase 3: Ranking Approach

**Figure 6:**
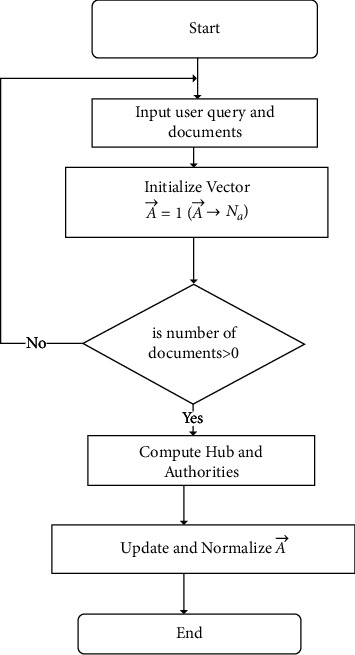
Proposed ranking approach.

In this phase, the researchers apply the ranking approach based on the study [19].  Figure 6 highlights the flow of processes in phase 3. Phase 3 initializes the vector and computes Hub and authorities similar to the HITS algorithm. However, a random walk feature is employed for updating Hub and authority weights.

**Figure 7:**
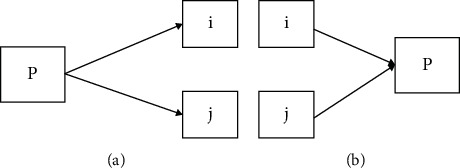
(a) Hub and (b) authority assignments.

The approach is the combination of PageRank [20], HITS [21], and SALSA [22] algorithms. It is a link-based ranking technique. Assume *a*_*i*_ be the authority weight, *h*_*i*_ be the hub weight. This ranking approach considers the document with higher *a*_*i*_ as better authorities and higher *h*_*i*_ as better hubs. Figures 7(a) and 7(b) show the authorities and Hub pointing with *P*. The weights of *h*_*i*_ and *a*_*i*_ are updated dynamically.

Documents are ranked according to the user query based on the weights of *h*_*i*_ and *a*_*i*_. It works similar to HITS using bipartite graph (*G*) and seed set (*R*_*f*_). In addition, the *P*-norms, a parameter, assign multiple normalized weights to each document link. A duplicative feature is employed to initiate Hub and authority, and vice-versa. The random walk feature of SALSA is used to identify the highly reachable node in *G*. Finally, normalization of the $\overset{⟶}{A}$ generates the ranked documents. The following procedure is applied for the ranking documents:

  Step 1: Input user query and initialize the *N*_*h*_ and *N*_*a*_ node and the parameter (*P*), P-norm value.
  Step 2: Initialize $\overset{⟶}{A}=1$ ($\overset{⟶}{A}\;⟶N_{a}$)
  Step 3: For each element *i* in *N*_*h*_
  Step 3a: For each element I in the set of nodes pointed by *i*^th^ node
  Compute Temp=Temp+ *a*_*j*_*P*/|*B*(*j*)|
  Step 3b: Compute $h_{j}=\sqrt[{P}]{\text{Temp}}$
  Step 4: For each element *k* in *N*_*a*_
  Step 4a: For each element *l* in *B*(*k*)
  Compute Temp=Temp+*h*_*l*_*P*/|*F*(*l*)|
  Step 4b: Compute $a_{k}=\sqrt[{P}]{\text{Temp}}$
  Step 5: Repeat Step 3 to 5 until weight converges
  Step 6: Update $\overset{⟶}{A}$ with authority weight
  Step 7: Normalize $\overset{⟶}{A}$, ranked documents.

### 3.4. Phase 4: Performance Evaluation

Phase 4 evaluates the ontological framework using the benchmark metrics. Precision, Recall, *F*1-measure, and Accuracy are the widely used metrics to measure the performance of IR systems. The following terms are applied in the evaluation metrics to ensure the effectiveness of the outcome generated by the frameworks.

True Positive (TP): The number of correctly predicted positive documents.

True Negative (TN): The number of correctly predicted negative documents.

False Positive (FP): The number of incorrectly predicted positive documents.

False Negative (FN): The number of incorrectly predicted negative documents.

Based on the above terms, the metrics are computed as follows:

Precision is a set of retrieved documents relevant to the user query.

(11) $$\begin{array}{c} \text{Precision}=\frac{\text{Number of relevant documents }\cap \text{Number of retrieved documents}}{\text{Number of retrieved documents}},\\ \text{Precision}=\;\frac{\text{TP}}{\text{TP}+\text{FP}}. \end{array}$$

It returns the number of documents divided by the number of retrieved documents. It can be computed for the topmost retrieved documents. For instance, Precision @10 indicates the top 10 retrieved documents.

The recall is a set of retrieved relevant documents. In other words, it is a number of documents divided by the number of relevant documents.

(12) $$\begin{array}{c} \text{Recall}=\;\frac{\text{Number of relevant documents}\cap \text{Number of retrieved documents}}{\text{Number of relevant documents}},\\ \text{Recall}=\;\frac{\text{TP}}{\text{TP}+\text{FN}}. \end{array}$$

*F*1–score is the harmonic mean of Precision and Recall.

(13) $$\begin{array}{c} F1-\text{score}=2*\;\left(\frac{\text{Precision}*\text{Recall}}{\text{Precision}+\text{Recall}}\right). \end{array}$$

Accuracy is the number of retrieved documents for a user query.

(14) $$\begin{array}{c} \text{Accuracy}=\;\frac{\text{TP}+\text{TN}}{\text{TP}+\text{TN}+\text{FP}+\text{FN}}. \end{array}$$

*R*–precision is used to ensure that the returned documents are relevant to a user query. It computes the recall value at *R*^th^ position.

Mean Average Precision (MAP) is the average precision for each user query.

MAP=∑_*q*=1_^*n*^Average Precision(*q*)/*n* where n is the number of queries (q).

## 4. Results and Discussion

**Figure 8:**
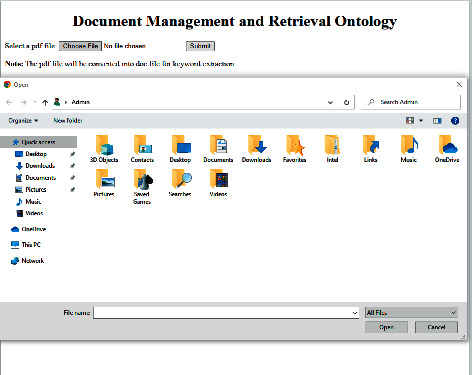
Document conversion and extraction interface.

To evaluate the performance of the proposed ontological framework (POF), a testbed containing 77 documents in PDF form is developed. Python 3.9.12 in Windows 10 professional environment is utilized for implementing the frameworks. Initially, a text extractor is employed to extract the text from the PDF.  Figure 8 illustrates the application interface for uploading the PDF file to convert it to a word file and extract key terms.

An Arabic dictionary is integrated with the text extractor to extract the Arabic content. MNB is used for building the ontology by classifying the documents with NER. Finally, the LBR method is applied for ranking the documents according to the user query.  Table 1 outlines the Arabic and English queries for evaluating the framework's performance. It comprises the five frequently used queries by the organizations to retrieve the documents.

**Figure 9:**
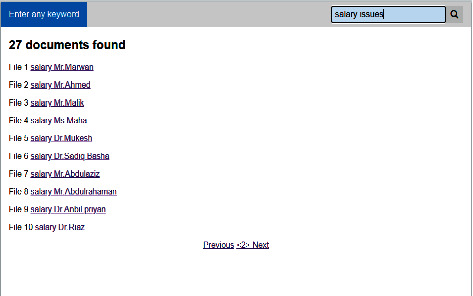
Results window.

Figure 9 shows the list of documents for the term “salay issues.” POF searches the documents and retrieves 27 documents based on the key terms. Using the hyperlink, the user can view the specific document.

Table 2 reports the findings of the performance evaluation of the POF. It outlines that the POF achieved compelling results. For instance, in Precision@77 for English Query 1, the POF offered Precision, Recall, *F*1-Score, and Accuracy of 97.3%, 97.1%, 97.2%, and 98.3. Similarly, in Precision@77 for Arabic Query, the POF presented Precision, Recall, *F*1-Score, and Accuracy of 97.7%, 98.4%, 98.05%, and 98.1%. It is evident from the outcome that the POF has produced a similar set of results for English and Arabic queries, respectively. The NER classification and link-based ranking approach have supported the POF in retrieving an optimal set of documents for user queries.

**Figure 10:**
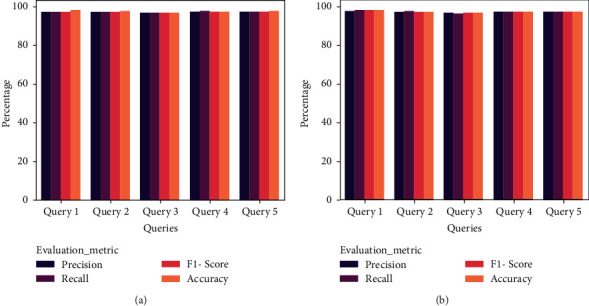
Performance analysis of POF (a) English (b) Arabic.

Figure 10 highlights the POF's overall performance (Precision @77) for the English and Arabic queries. The POF achieved an average *F*1-Score of 97% for five English and Arabic queries. It is noticed that the POF retrieved relevant documents for Arabic queries. Thus, it can support Saudi organizations in extracting effective results for employees and stakeholders.  Table 3 presents the findings of the comparative analysis of the ontological frameworks.

**Figure 11:**
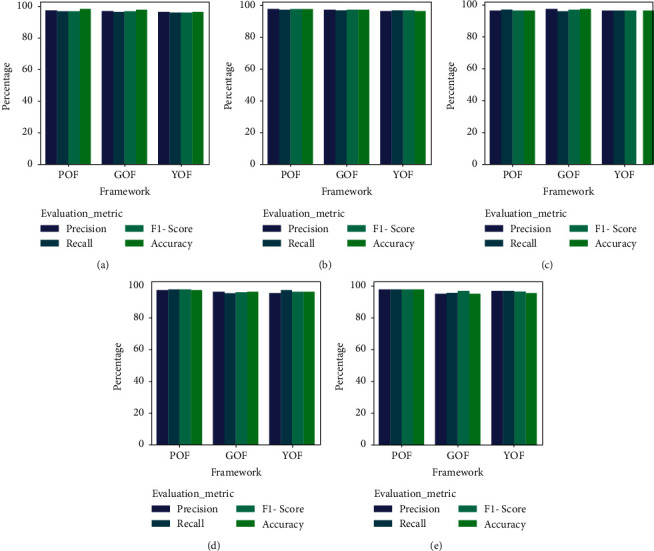
Comparative analysis of frameworks for English (a) query 1, (b) query 2, (c) query 3, (d) query 4, and (e) query 5.

**Figure 12:**
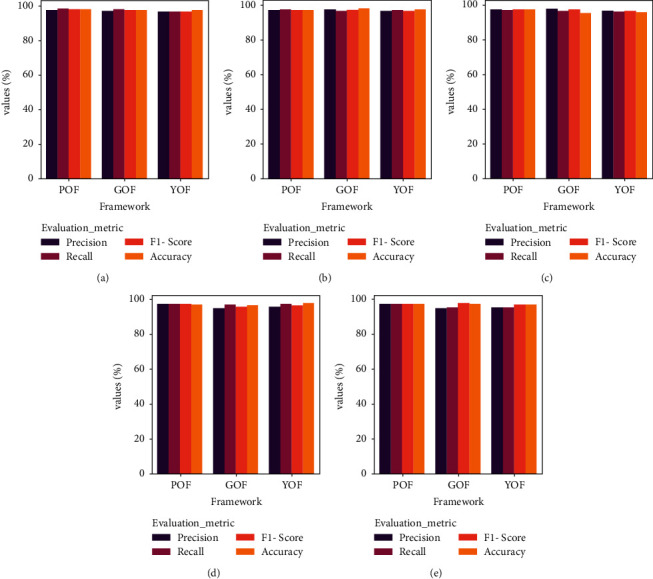
Comparative analysis of frameworks for Arabic (a) query 1, (b) query 2, (c) query 3, (d) query 4, and (e) query 5.

The frameworks have produced a better outcome for both English and Arabic queries, respectively. For context, the POF presented Precision, Recall, *F*1-Score, and Accuracy of 97.3%, 97.1%, 97.2%, and 98.3%, whereas the GOF and YOF have achieved Precision, Recall, *F*1-Score, and Accuracy of 97.1%, 96.4%, 96.75%, and 97.8% and 96.4%, 96.1%, 96.25%, and 96.4%. In addition, Figure 11 portrays the performance of the ontological framework for English queries, while Figure 12 presents the outcome for Arabic queries.

Figure 11 portrays the comparative analysis of the frameworks for the English queries. It represents that the POF has gained better Precision, Recall, *F*1-Score, and accuracy. Similarly, the GOF and YOF have accomplished higher Precision, Recall, *F*1-Score, and accuracy.

Likewise, Figure 12 presents the results for the Arabic queries. The frameworks have achieved a better result. However, the POF's overall performance is better than the existing frameworks. In addition to the benchmark metrics, Table 4 reveals the findings of *R*-Precision and MAP analysis. The POF outperforms both GOF and YOF, respectively. For instance, the value of *R*-Precision and MAP of the POF for Query 1 is 98.4% and 98.2, whereas GOF and YOF have offered R-Precision and MAP of 97.5% and 96.4% and 95.6% and 95.8%, respectively. The features of HITS and SALSA have favored the POF to retrieve a compelling set of documents compared to other frameworks.

**Figure 13:**
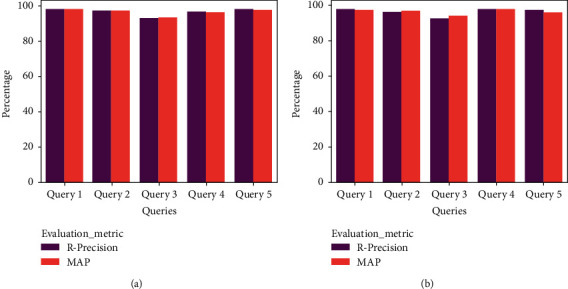
R-Precision and MAP analysis of POF (a) English and (b) Arabic.

**Figure 14:**
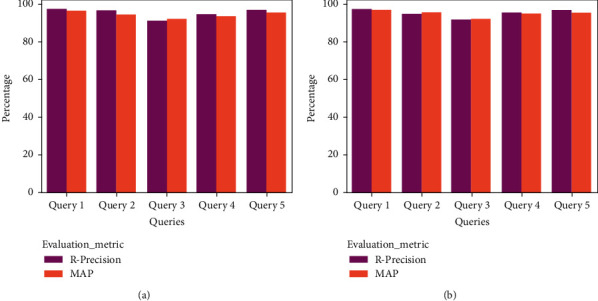
R-Precision and MAP analysis of GOF (a) English and (b) Arabic.

**Figure 15:**
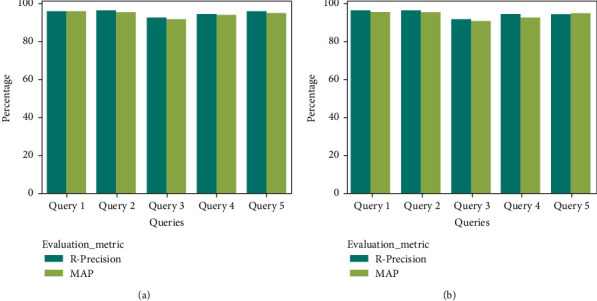
R-Precision and MAP analysis of YOF (a) English and (b) Arabic.

Figure 13 shows that the POF offered a supreme outcome for the English and Arabic queries compared to the GOF (Figure 14) and the YOF (Figure 15). It reveals that the effectiveness of data extraction, NER classification, and ranking approach supported the proposed framework to produce better results.

POF achieves a better Precision, Recall, *F*1-score, and Accuracy for both Arabic and English languages, respectively. It can be applied in any kind of document management environment. However, GOF and YOF are the ontological frameworks for specific documents which cannot be applied for general applications. In addition, POF offers a ranking technique for searching a bilingual document rather than GOF and YOF. It is a link-based searching technique, whereas GOF and YOF rank the documents according to the user query and term frequencies of the document. Thus, POF enables an effective searching environment for users compared to GOF and YOF.

### 4.1. Applications of the Proposed Framework

The proposed ontological framework can be applied in the real-time document management and retrieval environment. It enables an opportunity for the users to retrieve relevant documents based on the keywords. In addition, it offers the following applications for society.

Digital library: Using the proposed framework, a large corpus of documents can be developed to support the organization in facilitating a digital library for the employees to share information and manage their routine tasks.

Chatbot: The advent of AI techniques leads to the development of the question-answering system (Chatbot service) for the employees and stakeholders of an organization. The proposed framework can support the developers in training and test the Chatbot applications. The NB classifier offers the relation-based documents which the Chatbot system can use to provide relevant answers for the user queries.

Recommender system: Using phases 1 and 2, a recommender system can be developed for the employees to furnish useful data during document creation. The documents' data can be used as a keyword or metadata to search a document.

Furthermore, the bilingual feature of the proposed ontology supports Arabic and English-speaking users to share information effectively. It assists the user in overcoming the communication barrier and completing their routine tasks without difficulties.

## 5. Conclusion

This study developed an ontological framework for managing Arabic and English documents in Saudi Arabian organizations. The proposed framework comprises three phases for converting the PDF documents into ordinary word documents with a set of unique terms; a Naïve Bayes-based entity-relationship document classifier and a ranking technique for arranging documents as per the user query. The conversion technique uses a modified text extractor for extracting Arabic and English terms from the images. Furthermore, the entity-relationship technique arranges the document as per the relationship among the terms of the documents. The ranking technique combines the features of the HITS and SALSA ranking algorithm to rank the documents at a faster rate. A set of 77 documents were utilized to compare the performance of the proposed frameworks with the recent techniques. The outcome reveals that the proposed ontological framework achieves adequate Precision, Recall, *F*1-score, and Accuracy for the bilingual documents using a user query. In addition, it offers an effective bilingual document management environment for employees and stakeholders of Saudi Arabian organizations. The proposed framework can be extended to other languages. Furthermore, the ranking technique can be improved using metadata with the newer deep learning techniques.

## Figures and Tables

**Table 1:** User queries.

Queries	English	Arabic
1	What are the terms or words highly communicated by unit *A*?	ماهي الكلمة الأكثر استخداماً في الوحدة أ؟
2	What type of documents are accessed through the unit *B*?	ما نوع الوثائق التي يتم الوصول إليها من خلال الوحدة ب ؟
3	How many times unit *D* uses the term “center” in their communication?	كم مرة تستخدم الوحدة د مصطلح “مركز”في اتصالاتهم؟
4	What are the documents communicated by employee *A*?	ماهي الوثائق المرسلة من قبل الموظف أ؟
5	Who uses the word “delay” in the documents?	من يستخدم كلمة “تأخير في الوثائق”؟

**Table 2:** Performance analysis of the POF.

Queries	No of documents	English				Arabic			
		Precision	Recall	*F*1-score	Accuracy	Precision	Recall	*F*1-score	Accuracy
1	@10	98.2	97.8	98	98.2	98.1	97.6	97.85	98.3
	@30	97.4	97.6	97.5	97.6	97.5	97.4	97.45	97.6
	@50	97.5	98.3	97.9	98.1	97.6	97.7	97.65	97.9
	@77	97.3	97.1	97.2	98.3	97.7	98.4	98.05	98.1

2	@10	98.6	98.7	98.65	98.6	98.5	98.3	98.4	98.7
	@30	97.6	97.9	97.75	98.1	97.6	97.5	97.55	97.3
	@50	97.1	97.5	97.3	97.7	97.5	98.4	97.95	98.4
	@77	97.4	97.3	97.35	97.5	97.2	97.7	97.45	97.5

3	@10	98.2	98.4	98.3	98.6	98.4	98.6	98.5	98.7
	@30	97.9	97.2	97.55	97.9	97.5	97.6	97.55	97.1
	@50	97.4	97.6	97.5	97.5	97.3	97.4	97.35	97.3
	@77	96.7	96.8	96.75	96.7	96.8	96.5	96.65	96.8

4	@10	98.8	98.7	98.75	98.8	98.7	98.5	98.6	98.4
	@30	97.9	97.7	97.8	97.9	97.7	97.5	97.6	97.8
	@50	97.5	97.6	97.55	97.5	97.6	97.4	97.5	97.5
	@77	97.2	97.6	97.4	97.3	97.3	97.5	97.4	97.1

5	@10	98.6	98.8	98.7	98.6	98.5	98.6	98.55	98.7
	@30	97.7	97.5	97.6	97.7	97.6	97.3	97.45	97.5
	@50	97.1	97.3	97.2	97.6	97.3	97.6	97.45	97.7
	@77	97.3	97.4	97.35	97.5	97.2	97.4	97.3	97.1

**Table 3:** Comparative analysis of the frameworks.

Queries	Framework	English				Arabic			
		Precision	Recall	*F*1-score	Accuracy	Precision	Recall	*F*1-score	Accuracy
1	POF	97.3	97.1	97.2	98.3	97.7	98.4	98.05	98.1
	GOF	97.1	96.4	96.75	97.8	97.1	97.8	97.45	97.4
	YOF	96.4	96.1	96.25	96.4	96.7	96.8	96.75	97.5

2	POF	97.4	97.3	97.35	97.5	97.2	97.7	97.45	97.5
	GOF	97.2	96.8	97	97.1	97.5	96.7	97.1	98.1
	YOF	96.4	96.5	96.45	96.4	96.7	97.1	96.9	97.8

3	POF	96.7	96.8	96.75	96.7	96.8	96.5	96.65	96.8
	GOF	97.4	96.2	96.8	97.4	97.2	96.1	96.65	94.6
	YOF	96.4	96.5	96.45	96.4	96.1	95.7	95.9	95.3

4	POF	97.2	97.6	97.4	97.3	97.3	97.5	97.4	97.1
	GOF	96.2	95.3	95.75	96.4	94.7	97.1	95.88	96.4
	YOF	95.4	97.2	96.29	96.4	95.7	97.2	96.44	97.6

5	POF	97.3	97.4	97.2	97.5	97.2	97.4	98.05	97.1
	GOF	94.6	95.1	96.75	94.7	94.7	95.2	97.45	97.2
	YOF	96.5	96.5	96.25	95.2	95.1	95.3	96.75	96.8

**Table 4:** Findings of *R*-precision and MAP analysis.

Queries	Framework	English		Arabic	
		*R*-precision	MAP	*R*-precision	MAP
1	POF	98.4	98.2	97.6	96.8
	GOF	97.5	96.4	97.2	96.4
	YOF	95.6	95.8	96.3	95.3

2	POF	97.5	97.2	95.9	96.3
	GOF	97.1	94.4	94.8	95.2
	YOF	96.3	95.1	96.4	95.3

3	POF	93.2	93.4	92.1	93.5
	GOF	91.2	92.4	91.5	91.7
	YOF	92.4	91.5	91.6	90.8

4	POF	96.8	96.2	97.3	97.6
	GOF	94.6	93.7	95.1	94.6
	YOF	94.3	93.8	94.6	92.5

5	POF	98.3	97.6	97.1	95.8
	GOF	96.7	95.6	96.4	95.1
	YOF	95.6	94.8	94.3	95.1

## Data Availability

The data supporting the results can be available on request to the corresponding author.

## Supplementary Materials

Supplementary Materials The file contains the code for implementing the ontology. After implementing the ontology, we have to train and test with the dataset. The necessary code is mentioned in the article and included as an attachment.
