# Supplementary material for: Managing and Retrieving Bilingual Documents Using Artificial Intelligence-Based Ontological Framework
Source: Comput Intell Neurosci. 2022 Aug 25;2022:4636931. doi: 10.1155/2022/4636931 (PMC9436537; doi:10.1155/2022/4636931)
Supplement: Supplementary Materials — The file contains the code for implementing the ontology. After implementing the ontology, we have to train and test with the dataset. The necessary code is mentioned in the article and included as an attachment. [file 4636931.f1.docx]

| Import tweepy |  |
| --- | --- |
|  | import queue |
|  | import time |
|  | import pickle |
|  | import numpy as np |
|  | import scipy.sparse as sparse |
|  | from datetime import datetime as dt  from GOF import primary as pr  from YOF import primary as pr2  from evaluate import evaluation_method as evaluate |
|  | import sys |
|  |  |
|  | class Logger(): |
|  | interface |
|  | for logging |
|  | """ |
|  |  |
|  | def __init__(self, log_path, print_stdout=True, sep=' ', end='\n'): |
|  | """Initializes an instance of Logger |
|  |  |
|  | Args: |
|  | log_path: Path to the file to write the logs to |
|  | print_stdout: True if the logs must be written to stdout |
|  | sep: string to be used to separate arguments of printing |
|  | end: string to be after the last argument of printing |
|  | """ |
|  | self._log_file = open(log_path, 'w') |
|  | self._print_stdout = print_stdout |
|  | self._sep = sep |
|  | self._end = end |
|  |  |
|  | def log(self, *args): |
|  | """Logs whatever is present in args with current date and time |
|  |  |
|  | Uses instance variables self._sep for separating elements of args and |
|  | self._end after the last element of args. Writes to the log file |
|  | self._log_file. If self._print_stdout is True, logs are also written to |
|  | stdout |
|  |  |
|  | Args: |
|  | args: List of elements to be logged |
|  | """ |
|  | to_print = str(dt.now()) + ': ' |
|  | for i in args: |
|  | to_print += self._sep + str(i) |
|  | self._log_file.write(to_print + self._end) |
|  | self._log_file.flush() |
|  | if self._print_stdout: |
|  | print(to_print, end=self._end) |
|  | sys.stdout.flush() |
|  |  |
|  | def __del__(self): |
|  | """Close the log file when no references to the instance remain |
|  | """ |
|  | self._log_file.close() |
|  |  |
|  | class DatasetFetcher(): |
|  |  |
|  |  |
|  |  |
|  |  |
|  | def __init__(self, key, secret, logger): |
|  | """Initializes an instance of DatasetFetcher |
|  |  |
|  | Args: |
|  | key: key to be used for authentication |
|  | secret: secret to be used for authentication |
|  | logger: An instance of Logger to be used for logging purposed by public |
|  | member functions |
|  | """ |
|  | auth = tweepy.AppAuthHandler(key, secret) |
|  | self._api = tweepy.API(auth, retry_count=5) |
|  | self._visited = None |
|  | self._graph = None |
|  | self._logger = logger |
|  |  |
|  | def _print_api_rem(self): |
|  | """Print remaining quota for friends listing and followers listing endpoints |
|  | """ |
|  | try: |
|  | temp = self._api.rate_limit_status() |
|  | except tweepy.RateLimitError: |
|  | self._logger.log('Rate limit API limit reached') |
|  | except Exception as e: |
|  | self._logger.log('API limit exception: ', repr(e)) |
|  | else: |
|  | self._logger.log('Friends endpoint remaining: ', |
|  | temp['resources']['friends']['/friends/list']['remaining']) |
|  | self._logger.log('Followers endpoint remaining: ', |
|  | temp['resources']['followers']['/followers/list']['remaining']) |
|  |  |
|  | def _handle_limit(self, cursor, documents): |
|  | """Handles rate limits given a cursor |
|  | """ |
|  | while True: |
|  | try: |
|  | yield cursor.next() |
|  | except tweepy.RateLimitError: |
|  | try: |
|  | reset_time = self._api.rate_limit_status()['resources'][ documents]['/' + documents + '/list']['reset'] |
|  | except tweepy.RateLimitError: |
|  | self._logger.log('Sleeping for', 15 * 60, 'seconds') |
|  | time.sleep(15 * 60) |
|  | except Exception as e: |
|  | self._logger.log('Unexpected exception thrown: ', repr(e)) |
|  | self._logger.log('Sleeping for', 15 * 60, 'seconds') |
|  | time.sleep(15 * 60) |
|  | else: |
|  | self._logger.log('Sleeping for', max(reset_time - time.time() + 1, 1), 'seconds') |
|  | time.sleep(max(reset_time - time.time() + 1, 1)) |
|  | except tweepy.TweepError as e: |
|  | self._logger.log('tweepy.TweepError: code:', repr(e)) |
|  | break |
|  |  |
|  |  |
|  | def get_dataset( |
|  | self, seed_user, friends_limit, followers_limit, limit, live_save, |
|  | users_path, adj_list_path): |
|  | """Obtain the dataset |
|  |  |
|  | Args: |
|  | seed_user: id/screen_name/name of the user to start the bfs with |
|  | friends_limit: Maximum number of friends to consider for each user |
|  | followers_limit: Maximum number of followers to consider for each user |
|  | limit: Maximum number of users to find friends and followers of |
|  | live_save: Whether to save computed data frequently |
|  | users_path: Path to the file where the users info will be stored |
|  |  |
|  | adj_list_path: |
|  | """ |
|  |  |
|  | # Each node has three possible states - |
|  | # unvisited, visited but not explored, explored |
|  |  |
|  | # each key-value pair is of the form |
|  | # id: {'name': '', 'screen_name': ''} |
|  | # serves two purposes - |
|  | # ids in this are those that are visited |
|  | # stores user info corresponding to each id |
|  | self._visited = {} |
|  |  |
|  | # each key-value pair is of the form |
|  | # id: {'friends': [], 'followers': []} |
|  | # set of ids in graph equal to set of ids in visited |
|  | self._graph = {} |
|  |  |
|  | # ids that have been visited (and hence their info is in visited dict) |
|  | # but not yet explored |
|  | boundary = queue.Queue() |
|  |  |
|  | # Initialise |
|  | seed_user = self._api.get_user(seed_user) |
|  | self._visited[seed_user.id] = { |
|  | 'name': seed_user.name, |
|  | 'screen_name': seed_user.screen_name |
|  | } |
|  | self._graph[seed_user.id] = { |
|  | 'friends': [], |
|  | 'followers': [] |
|  | } |
|  | boundary.put(seed_user.id) |
|  |  |
|  | # Explore users as long as the total number of visited users is less than |
|  | # limit |
|  | should_break = False |
|  | live_save_suffix = 0 |
|  | while True: |
|  | self._logger.log('') |
|  | self._print_api_rem() |
|  | user_id = boundary.get() |
|  | self._logger.log('Selected:', self._visited[user_id]['screen_name'], |
|  | ',', self._visited[user_id]['name'], ',', user_id) |
|  |  |
|  | # Find friends |
|  | self._logger.log('Finding friends..') |
|  | cnt = 0 |
|  | for friend in self._handle_limit( |
|  | tweepy.Cursor(self._api.friends, user_id=user_id).items(friends_limit), 'friends'): |
|  |  |
|  | cnt += 1 |
|  | self._graph[user_id]['friends'].append(friend.id) |
|  | if friend.id not in self._visited: |
|  | self._visited[friend.id] = { |
|  | 'name': friend.name, |
|  | 'screen_name': friend.screen_name |
|  | } |
|  | self._graph[friend.id] = { |
|  | 'friends': [], |
|  | 'followers': [] |
|  | } |
|  | boundary.put(friend.id) |
|  | if len(self._visited) >= limit: |
|  | should_break = True |
|  | break |
|  | self._logger.log('Found', cnt, 'friends') |
|  |  |
|  | if should_break: |
|  | break |
|  |  |
|  | # Find followers |
|  | self._logger.log('Finding followers..') |
|  | cnt = 0 |
|  | for follower in self._handle_limit( |
|  | tweepy.Cursor(self._api.followers, user_id=user_id).items(followers_limit), 'followers'): |
|  |  |
|  | cnt += 1 |
|  | self._graph[user_id]['followers'].append(follower.id) |
|  | if follower.id not in self._visited: |
|  | self._visited[follower.id] = { |
|  | 'name': follower.name, |
|  | 'screen_name': follower.screen_name |
|  | } |
|  | self._graph[follower.id] = { |
|  | 'friends': [], |
|  | 'followers': [] |
|  | } |
|  | boundary.put(follower.id) |
|  | if len(self._visited) >= limit: |
|  | should_break = True |
|  | break |
|  | self._logger.log('Found', cnt, 'followers') |
|  |  |
|  | self._logger.log('Latest save suffix: ', live_save_suffix % 2) |
|  | if live_save: |
|  | self.save_dataset(users_path + str(live_save_suffix % 2), adj_list_path + str(live_save_suffix % 2)) |
|  | live_save_suffix += 1 |
|  |  |
|  | if should_break: |
|  | break |
|  |  |
|  | # Number of visited users is now equal to limit. Now find friends and |
|  | # followers of visited but unexplored users. Among these, consider only |
|  | # those that have already been visited, thus not increasing the number |
|  | # of users visited |
|  | self._logger.log('') |
|  | self._logger.log('Boundary..') |
|  | while not boundary.empty(): |
|  | self._logger.log('') |
|  | self._print_api_rem() |
|  | user_id = boundary.get() |
|  | self._logger.log('Selected:', self._visited[user_id]['screen_name'], |
|  | ',', self._visited[user_id]['name'], ',', user_id) |
|  |  |
|  | # Find friends |
|  | self._logger.log('Finding friends..') |
|  | cnt = 0 |
|  | cnt2 = 0 |
|  | for friend in self._handle_limit( |
|  | tweepy.Cursor(self._api.friends, user_id=user_id).items(friends_limit), 'friends'): |
|  |  |
|  | cnt += 1 |
|  | if friend.id in self._visited: |
|  | cnt2 += 1 |
|  | self._graph[user_id]['friends'].append(friend.id) |
|  | self._logger.log('Found', cnt, 'friends') |
|  | self._logger.log('Used', cnt2, 'friends') |
|  |  |
|  | # Find followers |
|  | self._logger.log('Finding followers..') |
|  | cnt = 0 |
|  | cnt2 = 0 |
|  | for follower in self._handle_limit( |
|  | tweepy.Cursor(self._api.followers, user_id=user_id).items(followers_limit), 'followers'): |
|  |  |
|  | cnt += 1 |
|  | if follower.id in self._visited: |
|  | cnt2 += 1 |
|  | self._graph[user_id]['followers'].append(follower.id) |
|  | self._logger.log('Found', cnt, 'followers') |
|  | self._logger.log('Used', cnt2, 'followers') |
|  |  |
|  | self._logger.log('Latest save suffix: ', live_save_suffix % 2) |
|  | if live_save: |
|  | self.save_dataset(users_path + str(live_save_suffix % 2), |
|  | adj_list_path + str(live_save_suffix % 2)) |
|  | live_save_suffix += 1 |
|  |  |
|  | self._logger.log('Queue size:', boundary.qsize()) |
|  |  |
|  | def save_dataset(self, users_path, adj_list_path): |
|  | """Save the dataset obtained by get_dataset |
|  |  |
|  | Args: |
|  | users_path: Path to the file where users info will be stored |
|  | adj_list_path: Path to the file where the adjacency list will be stored |
|  | """ |
|  | if users_path != '': |
|  | with open(users_path, mode='wb') as f: |
|  | try: |
|  | pickle.dump(self._visited, f) |
|  | except Exception as e: |
|  | self._logger.log('adjException:', repr(e)) |
|  |  |
|  | if adj_list_path != '': |
|  | with open(adj_list_path, mode='wb') as f: |
|  | try: |
|  | pickle.dump(self._graph, f) |
|  | except Exception as e: |
|  | self._logger.log('dump Exception:', repr(e)) |
|  |  |
|  | class ListToMatrixConverter(): |
|  | """An instance of ListToMatrixConverter is used to convert the data obtained |
|  | by the dataset fetcher from adjacency list form to a matrix form (and an |
|  | index-to-userid map) |
|  | """ |
|  |  |
|  | def __init__(self, adj_list_path): |
|  | """Initializes an instance of ListToMatrixConverter |
|  |  |
|  | Args: |
|  | adj_list_path: Path to the file where the adjacency list is stored |
|  | """ |
|  | with open(adj_list_path, 'rb') as f: |
|  | self._adj_list = pickle.load(f) |
|  | self._link_matrix = None |
|  | self._index_id_map = None |
|  |  |
|  | def convert(self): |
|  | """Use the adjacency list to create the link matrix and a dictionary that |
|  | maps the index in the link matrix to a user id |
|  | """ |
|  |  |
|  | # Put contents of self._adj_list in a matrix |
|  | size = len(self._adj_list) |
|  | self._link_matrix = np.zeros((size, size), dtype=np.int) |
|  |  |
|  | # Create map to save some time |
|  | id_index_map = {} |
|  | index = 0 |
|  | for user_id in self._adj_list: |
|  | id_index_map[user_id] = index |
|  | index += 1 |
|  |  |
|  | for user_id in self._adj_list: |
|  | for friend_id in self._adj_list[user_id]['friends']: |
|  | self._link_matrix[id_index_map[user_id], id_index_map[friend_id]] = 1 |
|  | for follower_id in self._adj_list[user_id]['followers']: |
|  | self._link_matrix[id_index_map[follower_id], id_index_map[user_id]] = 1 |
|  |  |
|  | self._index_id_map = {} |
|  | for i in id_index_map: |
|  | self._index_id_map[id_index_map[i]] = i |
|  |  |
|  | def save(self, map_path, link_matrix_path, use_sparse=False): |
|  | """Saves the map and link matrix created using the convert function |
|  |  |
|  | Args: |
|  | map_path: Path to the file where the map from link matrix index to |
|  | user id is to be stored |
|  | link_matrix_path: Path to the file where the link matrix is to be stored |
|  | use_sparse: True if the link matrix is to be stored as a sparse matrix |
|  | """ |
|  | if map_path != '': |
|  | with open(map_path, 'wb') as f: |
|  | try: |
|  | pickle.dump(self._index_id_map, f) |
|  | except Exception as e: |
|  | self._logger.log('Exception:', repr(e)) |
|  |  |
|  | if link_matrix_path != '': |
|  | with open(link_matrix_path, mode='wb') as f: |
|  | if use_sparse: |
|  | try: |
|  | sparse.save_npz(f, sparse.csr_matrix(self._link_matrix)) |
|  | except Exception as e: |
|  | self._logger.log('Exception:', repr(e)) |
|  | else: |
|  | try: |
|  | np.save(f, self._link_matrix) |
|  | except Exception as e: |
|  | self._logger.log('Exception:', repr(e)) |
|  |  |
|  |  |
|  | def main(): |
|  |  |
|  | key = 'j5idDIRvUfwI1213Nr14Drh33' |
|  | secret = 'jOw1Dgt8dJlu4rPh3GeoGofnIV5VKLkZ8fOQqYk1zUsaSMJnVl' |
|  | seed_user = 'Genius1238' |
|  |  |
|  | log_path = 'logs.txt' |
|  |  |
|  | users_path = '../data/users' |
|  | adj_list_path = '../data/adj_list' |
|  | map_path = '../data/map' |
|  | dense_link_matrix_path = '../data/dense_link_matrix' |
|  | sparse_link_matrix_path = '../data/sparse_link_matrix' |
|  |  |
|  | users_temp_path = '../data/temp/users_' |
|  | adj_list_temp_path = '../data/temp/adj_list_' |
|  |  |
|  | friends_limit = 200 |
|  | followers_limit = 200 |
|  | limit = 500 |
|  |  |
|  | logger = Logger(log_path) |
|  |  |
|  | # Fetch the dataset, store info of all users and store the adjacency list |
|  | app = DatasetFetcher(key, secret, logger) |
|  | logger.log('Obtaining dataset..') |
|  | app.get_dataset( |
|  | seed_user, friends_limit, followers_limit, limit, True, users_temp_path, |
|  | adj_list_temp_path) |
|  | logger.log('Dataset obtained') |
|  | app.save_dataset(users_path, adj_list_path) |
|  |  |
|  | # Create the link matrix and map using the adjacency list created |
|  | # previously and save them |
|  | c = ListToMatrixConverter(adj_list_path) |
|  | c.convert() |
|  | c.save(map_path, dense_link_matrix_path, use_sparse=False) |
|  |  |
|  | c = ListToMatrixConverter(adj_list_path) |
|  | c.convert() |
|  | c.save(map_path, sparse_link_matrix_path, use_sparse=True) |
|  | logger.log('Dataset Saved')  evaluate(pr,pr2,app) |
|  |  |
|  | if __name__ == '__main__': |
|  | main() |

Graph rendering

import matplotlib.pyplot as plt

import seaborn as sns

import pandas as pd

df1 = pd.read_csv("data_met.csv")

plt.figure(figsize=(11,4))

plt.subplot(1,2,1)

# the size of A4 paper

mm = sns.barplot(x = 'Queries', y = 'values_E', hue = "metric", data = df1, palette = 'magma')

mm.get_legend().set_visible(False)

mm.set_title("(a)",{"size":14})

mm.set_xlabel("Queries")

mm.set_ylabel("Percentage")

plt.subplot(1,2,2)

mn = sns.barplot(x = 'Queries', y = 'values_A', hue = "metric", data = df1, palette = 'magma')

mn.set_title("(b)",{"size":14})

mn.set_xlabel("Queries")

mn.set_ylabel("Percentage")

plt.legend(bbox_to_anchor=(1.05,1), title="Evaluation_metric")

Graph 2

import matplotlib.pyplot as plt

import seaborn as sns

df = pd.read_csv("data_met1.csv")

df1=df[df['Queries']=="Query 1"]

df2=df[df['Queries']=="Query 2"]

df3=df[df['Queries']=="Query 3"]

df4=df[df['Queries']=="Query 4"]

df5=df[df['Queries']=="Query 5"]

plt.figure(figsize=(14,9))

plt.subplot(2,3,1)

# the size of A4 paper

mm = sns.barplot(x = 'Framework', y = 'values_E', hue = "metric", data = df1, palette = 'viridis')

mm.get_legend().set_visible(False)

mm.set_title("(a)",{"size":14})

mm.set_xlabel("Framework")

mm.set_ylabel("Percentage")

plt.subplot(2,3,2)

# the size of A4 paper

mm = sns.barplot(x = 'Framework', y = 'values_E', hue = "metric", data = df2, palette = 'viridis')

mm.get_legend().set_visible(False)

mm.set_title("(b)",{"size":14})

mm.set_xlabel("Framework")

mm.set_ylabel("Percentage")

plt.subplot(2,3,3)

# the size of A4 paper

mn = sns.barplot(x = 'Framework', y = 'values_E', hue = "metric", data = df3, palette = 'viridis')

mn.set_title("(c)",{"size":14})

mn.set_xlabel("Framework")

mn.set_ylabel("Percentage")

plt.legend(bbox_to_anchor=(1.05,1), title="Evaluation_metric")

plt.subplot(2,3,4)

# the size of A4 paper

mm = sns.barplot(x = 'Framework', y = 'values_E', hue = "metric", data = df4, palette = 'viridis')

mm.get_legend().set_visible(False)

mm.set_title("(d)",{"size":14})

mm.set_xlabel("Framework")

mm.set_ylabel("Percentage")

plt.subplot(2,3,5)

# the size of A4 paper

mm = sns.barplot(x = 'Framework', y = 'values_E', hue = "metric", data = df5, palette = 'viridis')

mm.get_legend().set_visible(False)

mm.set_title("(e)",{"size":14})

mm.set_xlabel("Framework")

mm.set_ylabel("Percentage")

Graph 3

import matplotlib.pyplot as plt

import seaborn as sns

df = pd.read_csv("data_met1.csv")

df1=df[df['Queries']=="Query 1"]

df2=df[df['Queries']=="Query 2"]

df3=df[df['Queries']=="Query 3"]

df4=df[df['Queries']=="Query 4"]

df5=df[df['Queries']=="Query 5"]

plt.figure(figsize=(15,10))

plt.subplot(2,3,1)

# the size of A4 paper

mm = sns.barplot(x = 'Framework', y = 'values_A', hue = "metric", data = df1, palette = 'inferno')

mm.get_legend().set_visible(False)

mm.set_title("(a)",{"size":14})

mm.set_xlabel("Framework")

mm.set_ylabel("values(%)")

plt.subplot(2,3,2)

# the size of A4 paper

mm = sns.barplot(x = 'Framework', y = 'values_A', hue = "metric", data = df2, palette = 'inferno')

mm.get_legend().set_visible(False)

mm.set_title("(b)",{"size":14})

mm.set_xlabel("Framework")

mm.set_ylabel("values(%)")

plt.subplot(2,3,3)

# the size of A4 paper

mn = sns.barplot(x = 'Framework', y = 'values_A', hue = "metric", data = df3, palette = 'inferno')

mn.set_title("(c)",{"size":14})

mn.set_xlabel("Framework")

mn.set_ylabel("values(%)")

plt.legend(bbox_to_anchor=(1.05,1), title="Evaluation_metric")

plt.subplot(2,3,4)

# the size of A4 paper

mm = sns.barplot(x = 'Framework', y = 'values_A', hue = "metric", data = df4, palette = 'inferno')

mm.get_legend().set_visible(False)

mm.set_title("(d)",{"size":14})

mm.set_xlabel("Framework")

mm.set_ylabel("values(%)")

plt.subplot(2,3,5)

# the size of A4 paper

mm = sns.barplot(x = 'Framework', y = 'values_A', hue = "metric", data = df5, palette = 'inferno')

mm.get_legend().set_visible(False)

mm.set_title("(e)",{"size":14})

mm.set_xlabel("Framework")

mm.set_ylabel("values(%)")

import matplotlib.pyplot as plt

import seaborn as sns

df = pd.read_csv("data_met2.csv")

df1=df[df['Framework']=="POF"]

plt.figure(figsize=(11,4))

plt.subplot(1,2,1)

# the size of A4 paper

mm = sns.barplot(x = 'Queries', y = 'values_E', hue = "metric", data = df1, palette = 'magma')

mm.get_legend().set_visible(False)

mm.set_title("(a)",{"size":14})

mm.set_xlabel("Queries")

mm.set_ylabel("Percentage")

plt.subplot(1,2,2)

# the size of A4 paper

mn = sns.barplot(x = 'Queries', y = 'values_A', hue = "metric", data = df1, palette = 'magma')

mn.set_title("(b)",{"size":14})

mn.set_xlabel("Queries")

mn.set_ylabel("Percentage")

plt.legend(bbox_to_anchor=(1.05,1), title="Evaluation_metric")

import matplotlib.pyplot as plt

import seaborn as sns

df = pd.read_csv("data_met2.csv")

df1=df[df['Framework']=="GOF"]

plt.figure(figsize=(11,4))

plt.subplot(1,2,1)

# the size of A4 paper

mm = sns.barplot(x = 'Queries', y = 'values_E', hue = "metric", data = df1, palette = 'plasma')

mm.get_legend().set_visible(False)

mm.set_title("(a)",{"size":14})

mm.set_xlabel("Queries")

mm.set_ylabel("Percentage")

plt.subplot(1,2,2)

# the size of A4 paper

mn = sns.barplot(x = 'Queries', y = 'values_A', hue = "metric", data = df1, palette = 'plasma')

mn.set_title("(b)",{"size":14})

mn.set_xlabel("Queries")

mn.set_ylabel("Percentage")

plt.legend(bbox_to_anchor=(1.05,1), title="Evaluation_metric")

import matplotlib.pyplot as plt

import seaborn as sns

df = pd.read_csv("data_met2.csv")

df1=df[df['Framework']=="YOF"]

plt.figure(figsize=(11,4))

plt.subplot(1,2,1)

# the size of A4 paper

mm = sns.barplot(x = 'Queries', y = 'values_E', hue = "metric", data = df1, palette = 'rainbow')

mm.get_legend().set_visible(False)

mm.set_title("(a)",{"size":14})

mm.set_xlabel("Queries")

mm.set_ylabel("Percentage")

plt.subplot(1,2,2)

# the size of A4 paper

mn = sns.barplot(x = 'Queries', y = 'values_A', hue = "metric", data = df1, palette = 'rainbow')

mn.set_title("(b)",{"size":14})

mn.set_xlabel("Queries")

mn.set_ylabel("Percentage")

plt.legend(bbox_to_anchor=(1.05,1), title="Evaluation_metric")
